# The use of mesenchymal stromal cells in the treatment of coronavirus disease 2019

**DOI:** 10.1186/s12967-020-02532-4

**Published:** 2020-09-21

**Authors:** Maurice A. Canham, John D. M. Campbell, Joanne C. Mountford

**Affiliations:** Tissues, Cells & Advanced Therapeutics, Scottish National Blood Transfusion Service, The Jack Copland Centre, 52 Research Avenue North, Edinburgh, EH14 4BE UK

**Keywords:** COVID-19, SARS-CoV-2, Mesenchymal stromal cells, Acute respiratory distress syndrome

## Abstract

More than seven months into the coronavirus disease -19 (COVID-19) pandemic, infection from the severe acute respiratory syndrome coronavirus-2 (SARS-CoV-2) has led to over 21.2 million cases and resulted in over 760,000 deaths worldwide so far. As a result, COVID-19 has changed all our lives as we battle to curtail the spread of the infection in the absence of specific therapies against coronaviruses and in anticipation of a proven safe and efficacious vaccine. Common with previous outbreaks of coronavirus infections, SARS and Middle East respiratory syndrome, COVID-19 can lead to acute respiratory distress syndrome (ARDS) that arises due to an imbalanced immune response. While several repurposed antiviral and host-response drugs are under examination as potential treatments, other novel therapeutics are also being explored to alleviate the effects on critically ill patients. The use of mesenchymal stromal cells (MSCs) for COVID-19 has become an attractive avenue down which almost 70 different clinical trial teams have ventured. Successfully trialled for the treatment of other conditions such as multiple sclerosis, osteoarthritis and graft versus host disease, MSCs possess both regenerative and immunomodulatory properties, the latter of which can be harnessed to reduce the severity and longevity of ARDS in patients under intensive care due to SARS-CoV-2 infection.

## Background

The clinical condition known as coronavirus disease 2019 (COVID-19) has been brought to the fore of all human consciousness in the wake of this newly emergent and ongoing pandemic. Caused by the severe acute respiratory syndrome—coronavirus 2 (SARS-CoV-2), COVID-19 can manifest either asymptomatically, or generate symptoms ranging from persistent cough and fever, to severe respiratory failure and death [1, 2]. From its discovery during December 2019 in the Wuhan region of China, the rapid human to human and world-wide spread of the virus led the World Health Organisation (WHO) to declare COVID-19 a pandemic on the 11th of March 2020 [3–5].

With the lack of any specific treatment regimen for human coronavirus related diseases many different facets of biological and clinical research have been mobilised to explore various avenues of disease treatment, management and indeed eventual prevention through the creation of an effective vaccine [6–8]. However, due to the extensive lead time from the discovery of an effective and safe vaccine to its mass production and world-wide distribution, coupled with the observed asymptomatic/pre-symptomatic rapid spread of SARS-CoV-2, there is an urgent need for more immediate therapies for disease treatment and management [8, 9].

This review will focus on the emergence of SARS-CoV-2 and highlight the promising area of cell-based therapies as a potential avenue to treat the effects of this viral infection. Specifically, mesenchymal stromal cells (MSCs), which have been evaluated in previous pre-clinical and clinical studies for the treatments of various disease conditions due to their broad immunomodulatory, anti-inflammatory and regenerative capacities [10].

## Origins of SARS-CoV-2

A concentration of cases of a new respiratory disease, was first reported in the city of Wuhan in China during December 2019. Clinical examination of the patients revealed common symptoms of pneumonia, fever, reduced lymphocyte counts, pulmonary oedema and a lack of response to antibiotics, strongly suggesting a virally-induced disease. Epidemiological tracing of these early cases identified a common geographical origin to be a live animal market [1, 2]. Such symptoms and origins bore similar hallmarks to the outbreak of the severe acute respiratory syndrome (SARS) pandemic, also originating in China, during 2003 and caused by the SARS coronavirus (SARS-CoV) [11, 12]. Subsequent exploration of bronchoalveolar lavage fluid (BALF) from COVID-19 affected patients using deep meta-genomic sequencing revealed the aetiological agent to be a novel coronavirus that was eventually named SARS-CoV2 [1, 2, 13, 14].

SARS-CoV2 belongs to the beta coronavirus genera of the *Coronaviridae* family, which consist of enveloped, non-segmented, positive stranded RNA viruses. The receptor binding domain (RBD) of the spike (S) protein of coronaviruses is the principal determinant for its cellular tropism, with that of SARS-CoV-2 using the membrane bound angiotensin converting enzyme 2 (ACE2) for cell entry [1, 15, 16]. Also used by SARs-CoV [17], ACE2 is widely expressed in the upper and lower respiratory tract as well as in the lining of the intestines, the endothelia of the blood vessels and the heart [18, 19]. A separate receptor, dipeptidyl peptidase 4 (DPPA4), is used by the pathogenic Middle East Respiratory Syndrome coronavirus (MERS-CoV) [20], which has been responsible for the outbreak of potentially fatal pneumonia cases in Saudi Arabia since 2012 [21].

As for the origins of SARS-CoV-2, homology modelling has shown that the RBD of the spike protein is closely related to SARS-CoV [13, 22]. At the whole genome level, however, phylogenetic alignments have shown that SARS-CoV-2 was most similar to coronaviruses found within Chinese horseshoe bat populations [1, 13]. Previous studies have shown that SARS-CoV and MERS-CoV also have close sequence identities to coronavirus species circulating in bats [23, 24], and that bat coronaviruses can also use ACE2 as a receptor for cell entry in human cells [25]. These data strongly suggested that bats serve as an originating host for coronaviruses that can become zoonotic with the potential of causing human respiratory diseases. Previous examinations of the emergence potential of circulating bat coronaviruses to give rise to a new virus and ignite another pandemic concluded that such a scenario was almost inevitable and effectively predicted the current COVID-19 outbreak [26–28].

## Pathogenesis of COVID-19

Although most human coronavirus infections are mild, the outbreaks of SARS in 2003 and MERS in 2012 have caused more than 10,000 cumulative cases in the past two decades, with mortality rates of 10% for SARS-CoV and 37% for MERS-CoV [29, 30]. While we wait for the final tally of cases and overall mortality rate of COVID-19 to be determined, it is already known that SARS-CoV2 is extremely contagious as it can be transmitted asymptomatically [9]. From the first reported case in the city of Wuhan, COVID-19 rapidly spread to other regions of China, as well as reaching farther afield, with epicentres soon flourishing in Washington in the United States and the Lombardy region of northern Italy. Since then, the basic reproductive number (R0) has been shown to be as high as 2.5 [31]. As of the 16th August 2020, SARS-CoV-2 has been confirmed to have infected over 21.2 million people worldwide, across 212 countries and territories, leading to over 760,000 deaths [32]. Thus, in the first seven months since its onset, the death toll from COVID-19 is already many fold higher than any previously known coronavirus related disease.

The regions affected during the early stages of the COVID-19 emergence have reported varying numbers of patients requiring treatment in their intensive care units (ICU) upon admission to hospital with breathing difficulties, with percentages ranging between 5 and 32%. However, all centres have described common symptoms and features with their ICU patients including the presence of ground glass opacities in the lungs as observed by chest radiography, high neutrophil to lymphocyte ratio (lymphopenia) in the blood and the onset of acute respiratory distress syndrome (ARDS), with most patients requiring either invasive intubation or continuous positive airway pressure ventilation [4, 6, 33–35].

Disaggregation of fatality rates for different age groups has shown a consistent pattern across all countries of a significantly higher risk for elderly patients. Two separate studies have observed that the case fatality ratio rises steeply for those over 50 years of age and that the proportion of infected individuals requiring hospitalisation ranged from around 1% for the 20–29 age group to 18% for those aged 80 and upwards [36, 37]. Aside from the age effect, certain underlying conditions present in individuals can increase the chances of infection by SARS-CoV-2 and lead to poorer clinical outcome. From analyses undertaken from both within and outwith China the most prevalent comorbidities documented were hypertension, cardiovascular/cerebrovascular conditions and diabetes. Other notable higher risk groups included patients with cancer, chronic obstructive pulmonary disease and immunodeficiencies as well as patients who were smokers and recipients of transplants [38, 39].

ARDS is a common immunopathological feature of COVID-19, SARS and MERS and is brought about by an aggressive inflammatory response that can lead to respiratory arrest and death as seen in 70% of COVID-19 fatalities [40]. Initiated by viral infection of cells in the lower respiratory tract, local inflammation leads to the release of pro-inflammatory cytokines and chemokines which in turn leads to the recruitment of T cells and monocytes from the blood to the site of infection. This is evidenced by observations from several studies of lymphopenia in most COVID-19 patients as well as alterations in the counts of lymphocyte subsets in those with severe disease [6, 41–44]. The single case study of a 50-year-old patient who died from SARS-CoV-2 infection, reported a substantial reduction of circulating CD4 + and CD8 + T cells counts and relatively high proportions of HLA-DR + (CD4 + 3·47%) and CD38 + (CD8 + 39·4%) double-positive fractions, suggesting these cells were in a hyperactive state [41]. A more comprehensive investigation of 452 COVID-19 patients also revealed a reduction of total circulating CD4 T helper (Th) cells. With further sub-type analysis this report also showed an increase in proportion of naïve (CD3 + , CD4 + , CD45RA +) Th cells (44.5 vs 35.0%) and a decrease in numbers of (CD3 + , CD4 + , CD25 + , CD127low +) regulatory T cells (3.7 vs 4.5/μL) in severe cases when compared with non-severe cases [44]. Uncontrolled infiltration of inflammatory cells into the lungs generates an excessive release of proteases and reactive oxygen species that damages the alveolar epithelial-vascular endothelial barrier. Such damage leads to accumulation of fluid (oedema) in the alveoli and a reduction in the efficiency of gas exchange resulting in hypoxemia [45].

Aside from the local tissue damage and resultant ARDS imparted by the immune dysregulation in the lung, a systemic, uncontrolled inflammatory response may also result. Such a reaction, known as a cytokine storm, involves the release of potentially overwhelming amounts of pro-inflammatory cytokines and chemokines into the blood stream of COVID-19 patients including, but not limited to, IL-6, TNF-α, INF-γ, CXCL9 and CXCL10 [44, 46]. The systemic effect of this cytokine storm is septic shock which can lead to multiple organ failure and eventual death as observed in those severely affected by SARS-CoV-2 infection [4, 35, 46]. Transcriptional and serum profiling of COVID-19 patients in a recent study has revealed a unique and undesirable inflammatory response compared to other respiratory diseases. The reported signatures of markedly reduced type I and III INF responses identified a poor antiviral response to SARS-CoV2 compared to those from SARS-CoV or Influenza A viruses. This observation was coupled with measurements of elevated circulating chemokines and cytokines among COVID-19 patients, including IL-6, IL1RA, CCL2, CCL8 CXCL2, CXCL8, CXCL9, and CXCL16, indicating an enhancement of generalised inflammation [47].

Be it directly or indirectly linked to the effects of hyper-inflammation, another notable consequence of COVD-19 seen in a significant proportion of severely affected individuals is the impact on the coagulation system. Various preliminary investigations have shown an association of mortality among patients with increased circulating D-dimer levels, a marker of venous thromboembolism and deep vein thromboses. Two studies from the USA and the UK have reported overall thrombotic complication rates among COVID-19 patients of 9.5% and 7.7%, respectively [48, 49]. While standard anti-coagulation therapy with heparin has been administered, some patients appeared refractory to this intervention leading the authors to recommend urgent clinical trials exploring the role of anticoagulation treatments [49].

Several studies have now begun exploring the relationship of host genetics in relation to COVID-19 severity and progression. Previous explorations of other viral infections have shown that polymorphisms in human leukocyte antigen (HLA) and INF-induced transmembrane protein-3 loci influence susceptibility and severity of viral based respiratory disease [50]. A recent detailed report from the Severe COVID-19 Genome Wide Association Study Group, explored over 8.5 million single nucleotide polymorphisms between 1610 severely affected COVID-19 patients and 2205 control subjects, based in Spain and Italy. This work has uncovered two principal loci associated with susceptibility to respiratory failure. One of these loci, at chromosome 3p21.31, contained six genes, including the chemokine receptors CCR9 and CXCR6 which have functions in regulating the recruitment of tissue resident T cells, thus suggesting that disease susceptibility and severity can be affected by an inadequate immune response. The second associated signal, at chromosome 9q34.2 and coinciding with the ABO blood group locus, showed a higher risk in developing sever disease for patients with blood group A while those with blood group O were less susceptible [51].

While the prevalence for disease has been observed as equal among both sexes, males are at significantly higher risk for severe symptoms and death compared to females [52]. An exploration of the observed male bias in mortality since the onset of the COVID-19 pandemic may further highlight the role of the immune system on disease outcome when considering the influence of X-linked genes and the influence of sex hormones on immune responses [53]. The Toll-like receptor 7 gene (TLR7), responsible for innate viral sensing has been shown to escape X chromosome inactivation, thus leading to greater expression in female immune cells [54], while oestrogen can enhance production of T regulatory cells [55].

## Potential treatments

For the majority of COVID-19 patients thus far, treatment has consisted of supportive care coupled with measures to minimise the risk of viral transmission including the use of personal protective equipment and patient isolation. This supportive care for patients is typically the standard protocol because no specific antiviral therapies have yet been identified [8]. In one cohort of 138 patients in Wuhan, China, the antiviral drug oseltamivir, usually prescribed for the treatment of Influenza A and B, was administered to patients of COVID-19, in combination with antibacterial agents and in 45% of cases with corticosteroid medication to reduce inflammation. None of these treatment regimens proved effective [6]. More recently however, the use of the corticosteroid dexamethasone has been tested in the RECOVERY trial in which 2,300 patients in receipt of this immunosuppressant compared to 4,300 patients receiving standard care. The effect of dexamethasone has been shown to reduce fatality by 20% in patients requiring respiratory support [56]. However, this treatment had no effect on patients with milder symptoms and it has been suggested that its duration should be limited so as not to inhibit viral clearance [57].

Before the emergence of COVID-19, no specific treatments had been recommended for coronavirus related diseases. Numerous antivirals and other compounds normally used for the treatment of other ailments have shown some promise in their ability to curtail SARS-CoV and MERS-CoV replication using in vitro culture or animal models. Some examples include ribavirin for the treatment of respiratory syncytial virus, remdesivir, which was originally developed to treat Ebola and Marburg viruses, and lopinavir–ritonavir, approved by the United States Food and Drug Administration (US-FDA) to treat and prevent HIV/AIDS [58–60]. Randomised control trials, have been set up to assess the efficacy of lopinavir-ritonoavir in treating MERS [61, 62] and COVID-19 patients (ChiCTR2000029308). For compassionate use, a recently published study exploring Remdesivir as a treatment regimen for SARS-CoV2 infection, reported clinical improvement in 68% of 53 affected patients and randomised controlled trials clinical trials (NCT04257656, NCT04252664) are currently underway to explore this treatment regimen [62].

Other compounds targeting the host response to coronavirus infections have also been investigated for the treatment of coronavirus infections [63]. The serine protease inhibitor camostat mesylate, normally used to treat chronic pancreatitis, blocks activity of the cellular protease TMPRSS2 that is necessary to process SARS-CoV and SARS-CoV-2 spike protein for cell entry. While this compound was shown to be effective in reducing both SARS-CoV and SARS-CoV-2 entry in cell culture experiments, its suitability for treating patients severely affected by coronavirus related diseases has yet to be determined [64]. Furthermore, because coronaviruses can enter cells through the cathepsin-based endosomal pathway, by-passing the requirement for processing by TMPRSS2, the use of camostat mesylate may not be completely effective in preventing SARS-CoV-2 infection [58, 63]. Tocilizumab is an anti-IL6 humanised monoclonal antibody approved for the treatment of juvenile arthritis and has also been used to curtail the potentially life-threatening phenomenon of cytokine release syndrome that can occur following administration of CAR-T or immune checkpoint inhibitor therapies for cancer. Due to its immunomodulatory properties, several studies have explored its use for the treatment of COVID-19 patients, however, with mixed outcomes and inconsistent study designs, the benefits are inconclusive at this stage but do warrant further investigation [65–68]. The role of androgens has been postulated to explain the disparity of severe COVID-19 cases between men and women due to the presence of an androgen response element upstream of the gene encoding the TMPRSS2 protease that primes the S protein of SARS-CoV-2, facilitating infection. Observations of Spanish patients with COVID-19 have noted a substantial proportion of severely affected individuals having androgenetic alopecia and one preliminary study from Italy has found that prostate cancer patients receiving androgen deprivation therapy (ADT) were partially protected from SARS-CoV-2 infection [69–71].

Another promising route for treatment of COVID-19 is the application of convalescent plasma (CP) from recovering patients. Also known as passive antibody therapy, this method uses antibodies harvested from the plasma of previously infected patients who have subsequently recovered from disease symptoms and had been used during the 1918 Spanish Flu pandemic [72]. Previous exploration of CP therapy for coronavirus related disease had shown a positive benefit among Hong Kong patients with severe SARS in 2005. Critically ill patients who received CP within the first 14 days of infection were discharged from hospital earlier than control subjects [73]. Similar clinical benefits have been detailed in a meta-analysis study examining the use of CP therapy for viral-based respiratory diseases, including SARS, MERS and those caused by various influenza strains [74]. The effective antibody titre within CP for treating SARS patients was determined to be 1:61 using a neutralization assay, which was a measure of the ability of sera to neutralize the infectivity of SARS-CoV in cell culture [75]. Two preliminary reports from Chinese hospitals administering CP to cohorts of 5 and 10 patients with severe COVID-19 have so far noted promising outcomes for the majority of those undergoing the treatment with each study employing different amounts of neutralising antibody titres, 1:40 versus 1:160 [76, 77]. The latter of these titres matches that used in the previous successful treatment of influenza A with CP and is also the minimum neutralising antibody titre recommended recently by the US-FDA, while that recommended by the European Commission is 1:320 [74, 78]. More recently, in the US, a study evaluating CP therapy among 5,000 individuals with COVID-19 has reported the incidence of serious adverse events to be less than 1%, highlighting the safety of this treatment regimen [79]. As such, two framework clinical trials in the UK, the RECOVERY trial (NCT04381936) and the REMAP CAP trial (NCT02735707), have been set-up to include evaluation of CP treatment that shall be managed by NHS blood transfusion services.

## The properties of MSCs and their suitability for treating immune dysregulation in COVID-19

Since their discovery in the 1950s when they were first isolated from bone marrow (BM) and circulating blood, there has been a growing recognition that MSCs possess qualities that can promote tissue regeneration and suppress inflammation at sites of injury and disease [80]. The substantial body of knowledge that has been accrued over the years from numerous studies with in vitro and animal models has led to an ever increasing number of clinical trials being initiated to explore the utility of MSCs for a variety of disorders. The first in human trial was for patients of breast cancer recovering after high-dose chemotherapy treatment. This study demonstrated an accelerated reconstitution of the haematopoietic system in patients co-infused with autologous haematopoietic stem cells (HSCs) and MSCs [81]. Several years later, two separate multicentre trials showed benefits of MSC therapy in treating patients suffering from graft versus host disease (GvHD), following receipt of allogeneic HSCs [82, 83]. Canada, New Zealand and Japan have since issued approval for treatment of GvHD in children using MSCs, marketed under the name of Prochymal [10]. The benefits of MSCs have since been assessed for many other conditions in both pre-clinical and clinical trial settings. Notable examples include exploring the reduction of inflammation in patients with osteoarthritis [84], multiple sclerosis [85, 86], as well as their use in the treatment of type 1 diabetes mellitus either alone [87] or together with transplanted pancreatic islets to promote integration and survival [88].

MSCs are found in perivascular spaces throughout the body, forming niches in most tissues and providing quiescent support until their requirement to be mobilised to sites of inflammation or injury is signalled. As well as promoting healing through the enhancement of local vascularisation [88, 89], MSCs may themselves differentiate into various cell types in a cell replacement strategy to accomplish tissue repair [90]. Indeed, their potential to differentiate into adipocytes, osteocytes and chondrocytes in vitro is heralded as a principle criterion for their identity [91]. Their attractiveness for use in cell therapy stems from the fact that they can be isolated from various sources aside from BM (adipose tissue, dental pulp and the umbilical cord) as well as their straightforward and rapid expansion in cell culture, allowing large cell banks to be generated and cryopreserved for repeated therapeutic usage. Furthermore, with their low expression of major histocompatibility complex (MHC) type 1 and absence of MHC type 2 expression, MSCs are considered non-immunogenic, making them an ideal allogeneic cellular therapeutic [92].

Specific to their suitability for treating coronavirus infection and the manifest condition of ARDS is the ability of MSCs to restore a balanced immunological response at sites of inflammation by interaction of various components of the immune system and surrounding environment. A principal characteristic is their ability to interact with the innate and adaptive immune systems by sensing the inflammatory state of their local microenvironment and detecting the presence of microbes through stimulation of TLRs expressed on their surface. In the absence of any inflammatory signals (e.g. low TNF-α and INF-γ levels) or through the stimulation of TLR4 receptors by bacterial lipopolysaccharides, MSCs release pro-inflammatory signals (e.g. CXCL10 and IL6) to recruit natural killer cells and activated T cells. Conversely, in the presence of an inflammatory microenvironment (e.g. high TNF-α and INF-γ levels) or stimulation of TLR3 receptors by viral RNA, MSCs release PGE2, IDO1 and TGF-β as anti-inflammatory signals that can favour the emergence of both regulatory dendritic and T cells. These balances are kept in check by a delicate reciprocal interaction of MSCs and resident macrophages to ensure tissue homeostasis [93–95].

Finally, a further property of MSCs that makes them a suitable consideration for treating the effects of COVID-19 is the fact that following intravenous delivery into patients they tend to get caught in the capillary bed of the lungs [96]. As a result, not only can they reduce inflammation at the principal site of infection but they can also restore pulmonary vascular endothelial integrity and remove alveolar oedema fluid as shown for example in patients treated for *E.Coli* endotoxin-induced ARDS [97].

## MSCs in pre-clinical models and clinical investigations of acute lung injury

In several pre-clinical in vitro airway epithelial cell and mouse model studies, MSCs have been employed to explore their capacity in reducing the pathology of influenza virus-induced lung injury.

Administration of human BM-derived MSCs into 8–12 month old mice infected with the H5N1 strain of influenza has been shown to reduce weight loss, lung oedema, BALF inflammatory cytokine concentrations and fatality. The same study also demonstrated that MSCs cultured along with human alveolar epithelial cells infected with the same strain produce keratinocyte growth factor, which is important in the clearance of alveolar fluid build-up [98]. Another mouse model study examined the effectiveness of murine bone marrow derived MSCs in H9N2 infected mice. Those mice in receipt of MSCs following infection showed improved survival rates, lung histopathology and reduced serum and BALF inflammatory cytokine and chemokine concentrations compared to the control group [99]. While most studies explore the potential of BM-derived MSCs, a report comparing their performance versus umbilical cord (UC) derived MSCs in a human alveolar epithelial cell and mouse models of H5N1 infection demonstrated better restoration of alveolar fluid clearance and protein permeability with UC-MSCs [100].

In the clinical setting, there have been two recent notable completed studies evaluating the performance of MSCs as a therapeutic for ARDS. The first, known as the START study, was a phase 2a safety, prospective, double-blind, multicentre, randomised trial to assess treatment with one intravenous dose BM-MSCs of (10 × 10^6^ cells/kg) compared with placebo in patients with moderate to severe ARDS. While the overall success of this treatment was only marginal, there was a trend of improved oxygenation in the MSC group with no adverse reactions. It was suggested that post-thaw processing of the cryopreserved MSCs may have contributed to the modest clinical outcomes in the MSC group [101, 102]. The second clinical study of note examined the performance of menstrual blood-derived MSCs in for the treatment of 17 critically ill patients with H7N9 influenza induced ARDS. Those administered MSCs as a means to alleviate ARDS received either 3 or 4 infusions of 1 × 10^6^ cells/kg. Compared to the control group, which consisted of 44 patients receiving the standard care of treatment for severe influenza symptoms, the MSC group had a significantly reduced fatality rate survival outcome (54.5 versus 17.6%). No adverse events were noted after administration of MSCs and a 5-year follow-up of 4 of these patients has not revealed any long-term harmful effects. Due to the positive outcomes noted the authors of this study suggested MSC treatment of ARDS may be beneficial to patients of COVID-19 [103].

Other clinical trials underway utilising UC and BM-MSCs for the treatment of ARDS are listed in Table 1 together with those previously published. One ongoing trial, MUST-ARDS, is a joint US/UK multi-centre study administering ex vivo expanded BM-derived multipotent adult progenitor cells (MAPC). A preliminary report has noted that MAPC administration was well tolerated and patients receiving this treatment had better survival outcomes and fewer days with ventilator assistance compared to those receiving placebo [104].

**Table 1:** List of completed and ongoing clinical trials for the use of MSC in the treatment of ARDS

Trial ID	Title	Ref	Site	MSC source	No.of Pts	Route	Outcomes for MSC group
NCT01902082	Adipose-derived Mesenchymal Stem Cells in Acute Respiratory Distress Syndrome	[111]	China, Zhejiang	AT	6 Control 6 Exp	I.V	No adverse reactions. Modest clinical improvements
NCT02097641	Human Mesenchymal Stromal Cells For Acute Respiratory Distress Syndrome (START)	[99]	USA, multi-centre	BM	20 Control 40 Exp	I.V	No adverse reactions. Modest clinical improvements
ChiCTR-OCC-15006355	Clinical Study of Mesenchymal Stem Cell Treatment for Acute Respiratory Distress Syndrome Induced by Epidemic Influenza A (H7N9) Infection: A Hint for COVID-19 Treatment	[100]	China, Zhejiang	MB	44 Control 17 Exp	I.V	No adverse reactions. Reduction of Fatality rate
NCT02611609	A phase 1/2 study to assess MultiStem therapy in ARDS (MUST-ARDS)	[104]	USA, UK	BM-MAPC	9 Control 9 Exp 1 9 Exp 2 9 Exp 3	I.V	Preliminary: No adverse reactions. Reduction of Fatality rate. Reduction of ventilator assistance time
NCT02804945	Mesenchymal stem cells (MSCs) for treatment of ARDS (ARDS) in stem cell transplant patients		USA, Texas	BM	20 Exp	not stated	ongoing
NCT02215811	Treatment of severe acute respiratory distress syndrome with allogenic bone marrow derived MSCs		Sweden, Stockholm	BM	10 Exp	not stated	ongoing
NCT02444455	Human umbilical cord derived mesenchymal stem cell therapy in acute lung injury (UCMSC-ALI)		China, Beijing	UC	20 Exp	I.V	ongoing

*AT* adipose Tissue, *BM *bone marrow, *MAPC* multi adult progenitor cell, *MB* menstrual blood, *I.V.* intra venous, *Exp* experimental

## Treating COVID-19 patients with MSCs to alleviate ARDS

Despite the very recent emergence of the COVID-19 pandemic two pilot reports from Chinese hospitals have already been published documenting preliminary outcomes for patients receiving MSCs in attempts to curtail the effects of SARS-CoV-2 infection.

The first, a single-centre open-label pilot study has used MSCs, of unknown tissue origin, to treat seven patients with differing severities of ARDS resulting from COVID-19 in a Beijing Hospital. This small cohort included two patients with moderate symptoms, four with severe and one patient with critically severe disease requiring mechanical ventilation. Along with three severe case control patients receiving placebo, all patients were assessed for efficacy and safety outcomes after two weeks. No reactions to infusion nor delayed hypersensitivity were detected. All patients receiving MSCs showed clinical improvement after 2 days, with three of them (1 severe and 2 moderate) being discharged from hospital after 10 days, at the time of publication. Using mass cytometry for functional and phenotypic analysis of cell types present in patients’ blood before and after treatment and compared with the profile from normal individuals, the authors of the report noted an overall increase in circulating lymphocytes and an increase of CXCR3 negative regulatory T cells and Dendritic cells, in all severely affected patients in receipt of MSC therapy. For the critically severe case, there was a depletion of circulating activated pro-inflammatory CXCR4 positive CD4 T cells, CD8 T cells and NK cells. Additionally, in comparison to the placebo group, the patients receiving MSCs had decreased levels of C-reactive protein and TNF-α together with a concomitant increase in IL-10, further indicating a switch from a pro-inflammatory to an anti-inflammatory state. Finally, the authors undertook gene expression analysis using RNA-seq to define the properties of the MSCs administered to patients in this pilot study. They showed that their source of MSCs was negative for ACE2 and TMPRSS2 expression, strongly suggesting a natural resistance to infection by SARS-CoV-2, thus making this cellular therapeutic an attractive method of treating COVID-19 patients [105].

The second report, was a single case study, in which a 65 woman in China with worsening conditions due to SARS-CoV-2 induced ARDS, was treated with three doses of 5 × 10^7^ UC-MSCs. Following receipt of the second dose, significant clinical improvement was observed as noted by a reduction of the pneumonia detected in chest CT scans and invasive ventilation was no longer required. No adverse effects were noted and the clinical changes were accompanied by a return of normal neutrophil and lymphocyte counts, suggesting a systemic immunological benefit in this case study [106].

At the time of writing this review, many other clinical trials utilising MSCs have been initiated for the treatment of COVID-19, with 69 studies documented on the WHO International Clinical Trial Registry Platform [107]. Trials have now been registered in numerous countries: China, 27; USA, 11; Spain, 10; Iran, 9; Germany, 2; and one in each of Belarus, Brazil, Canada, Colombia, Denmark, Jordan, and the UK (Table 2). In relation to the tissue of origin for the derivation of MSCs to be administered, only 5 trials are utilising what is considered to be the gold standard for clinical use, BM-MSCs. UC-MSCs (including from Wharton’s Jelly) are the most common source with 27 trials, followed by 9 from adipose tissue, 4 from dental pulp, 3 from placenta, 1 from menstrual blood, 1 from mesenchyme-angioblasts and 1 from olfactory mucosa with 14 not declaring their origin [107]. In recent years, the benefits of and relative ease with which UC-MSCs can be isolated and maintained compared with BM-MSCs has been widely recognised and thus far they have been the most readily adopted cellular therapeutic treatment for COVD-19 [88, 108, 109]. Interestingly, there are 5 trials utilising either MSC conditioned media or exosomes containing components of the MSC secretome, believed to execute the immunomodulatory benefits which have been documented in various studies [45, 95, 110].

**Table 2:** List of ongoing clinical trials for exploring the use of MSCs or their derivatives in the treatment of COVID-19

Trial ID	Title	Site	Tissue Source	No. of Patients
ChiCTR2000029569	Safety and efficacy of umbilical cord blood mononuclear cells conditioned medium in the treatment of severe and critically novel coronavirus pneumonia (COVID-19): a randomized controlled trial	China, Hubei	Conditioned Media from Umbilical Cord MSCs	15 Control 15 Exp
ChiCTR2000030173	Key techniques of umbilical cord mesenchymal stem cells for the treatment of novel coronavirus pneumonia (COVID-19) and clinical application demonstration	China, Hu'nan	Umbilical Cord	30 Control 30 Exp
ChiCTR2000030116	Safety and effectiveness of human umbilical cord mesenchymal stem cells in the treatment of acute respiratory distress syndrome of severe novel coronavirus pneumonia (COVID-19)	China, Jiangxi	Umbilical Cord	16 Exp
NCT04269525	Umbilical Cord(UC)-Derived Mesenchymal Stem Cells(MSCs) Treatment for the 2019-novel Coronavirus(nCOV) Pneumonia	China, Hubei	Umbilical Cord	10 Exp
ChiCTR2000030138	Clinical Trial for Human Mesenchymal Stem Cells in the Treatment of Severe Novel Coronavirus Pneumonia (COVID-19)	China, Beijing	Umbilical Cord	30 Control 30 Exp
ChiCTR2000029990	Clinical trials of mesenchymal stem cells for the treatment of pneumonitis caused by novel coronavirus (COVID-19)	China, Beijing	Not Specified	60 Control 60 Exp
ChiCTR2000030088	Umbilical cord Wharton's Jelly derived mesenchymal stem cells in the treatment of severe novel coronavirus pneumonia (COVID-19)	China, Beijing	Umbilical Cord	20 Control 20 Exp
ChiCTR2000030020	The clinical application and basic research related to mesenchymal stem cells to treat novel coronavirus pneumonia (COVID-19)	China, Hu'nan	Not Specified	20 Exp
ChiCTR2000030261	A study for the key technology of mesenchymal stem cells exosomes atomization in the treatment of novel coronavirus pneumonia (COVID-19)	China, Jiangsu	MSC Exosomes (origin not specified)	13 Control 13 Exp
NCT04276987	A Pilot Clinical Study on Inhalation of Mesenchymal Stem Cells Exosomes Treating Severe Novel Coronavirus Pneumonia	China, Hubei	Adipose Tissue—MSC Exosomes	30 Exp
ChiCTR2000030484	HUMSCs and Exosomes Treating Patients with Lung Injury following Novel Coronavirus Pneumonia (COVID-19)	China, Hubei	Umbilical Cord + Exosomes	30 Control 30 Exp 1 30 Exp 2
ChiCTR2000029580	Severe novel coronavirus pneumonia (COVID-19) patients treated with ruxolitinib in combination with mesenchymal stem cells: a prospective, single blind, randomized controlled clinical trial	China, Hubei	MSCs (origin not specified) + Ruxolitinib	35 Control 35 Exp
ChiCTR2000030866	Open-label, observational study of human umbilical cord derived mesenchymal stem cells in the treatment of severe and critical patients with novel coronavirus pneumonia (COVID-19)	China, Hu'nan	Umbilical Cord	30 Exp
ChiCTR2000030835	Clinical study for the efficacy of Mesenchymal stem cells (MSC) in the treatment of severe novel coronavirus pneumonia (COVID-19)	China, He'nan	Umbilical Cord	10 Exp 1 10 Exp 2
NCT04302519	Novel Coronavirus Induced Severe Pneumonia Treated by Dental Pulp Mesenchymal Stem Cells	China, Hubei	Dental Pulp	24 Exp
NCT04313322	Treatment of COVID-19 Patients Using Wharton's Jelly-Mesenchymal Stem Cells	Jordan	Umbilical Cord	5 Exp
ChiCTR2000031319	Safety and Efficacy Study of Allogeneic Human Dental Pulp Mesenchymal Stem Cells to Treat Severe Pneumonia of COVID-19	China, Hubei	Dental Pulp	10 Control 10 Exp
ChiCTR2000031494	Clinical study for stem cells in the treatment of severe novel coronavirus pneumonia (COVID-19)	China, Hubei	Umbilical Cord	18 Control 18 Exp
ChiCTR2000031430	Clinical study of human umbilical cord mesenchymal stem cells in the treatment of novel coronavirus pneumonia (COVID-19) induced pulmonary fibrosis	China, Beijing	Umbilical Cord	100 Control 100 Exp
ChiCTR2000029606	Clinical Study for Human Menstrual Blood-Derived Stem Cells in the Treatment of Acute Novel Coronavirus Pneumonia (COVID-19)	China, Zhejiang	Menstrual Blood—MSCs ± artificial liver therapy	25 Control 18 Exp 1 10 Exp 2 10 Exp 3
NCT04252118	Mesenchymal Stem Cell Treatment for Pneumonia Patients Infected With COVID-19	China, Zhejiang	Umbilical Cord	10 Control 10 Exp
NCT04273646	Study of Human Umbilical Cord Mesenchymal Stem Cells in the Treatment of Severe COVID-19	China, Hubei	Umbilical Cord	24 Control 24 Exp
NCT04288102	Treatment With Mesenchymal Stem Cells for Severe Corona Virus Disease 2019(COVID-19)	China, Hubei	Not Specified	45 Control 45 Exp
NCT04315987	NestCell® Mesenchymal Stem Cell to Treat Patients With Severe COVID-19 Pneumonia (HOPE)	Brazil, Sao Paulo	Not Specified	66 Exp
NCT04336254	Safety and Efficacy Study of Allogeneic Human Dental Pulp Mesenchymal Stem Cells to Treat Severe COVID-19 Patients	China, Hubei	Dental Pulp	10 Control 10 Exp
NCT04339660	Clinical Research of Human Mesenchymal Stem Cells in the Treatment of COVID-19 Pneumonia	China, Hubei	Umbilical Cord	15 Control 15 Exp
NCT04341610	ASC Therapy for Patients With Severe Respiratory COVID-19 (ASC COVID-19)	Denmark, Copenhagen	Adipose Tissue	20 Control 20 Exp
NCT04345601	Mesenchymal Stromal Cells for the Treatment of SARS-CoV-2 Induced Acute Respiratory Failure (COVID-19 Disease)	USA, Texas	Bone Marrow	30 Exp
NCT04346368	Bone Marrow-Derived Mesenchymal Stem Cell Treatment for Severe Patients With Coronavirus Disease 2019 (COVID-19)	China, Guangdong	Bone Marrow	10 Control 10 Exp
NCT04348461	BAttLe Against COVID-19 Using MesenchYmal Stromal Cells	Spain, Madrid	Adipose Tissue	50 Control 50 Exp
NCT04349631	A Clinical Trial to Determine the Safety and Efficacy of Hope Biosciences Autologous Mesenchymal Stem Cell Therapy (HB-adMSCs) to Provide Protection Against COVID-19	USA, Texas	Adipose Tissue	56 Exp
EUCTR2019-002688-89-ES	Clinical Study to Assess the Safety and Preliminary Efficacy of HCR040 in Acute Respiratory Distress Syndrome	Spain, Bizkaia	Not Specified	14 Control 14 Exp
EUCTR2020-001682-36-ES	Treatment of COVID-19 with allogeneic mesenchymal cells (MSVÂ®)	Spain, Madrid	Not Specified	12 Control 12 Exp
EUCTR2020-001266-11-ES	Clinical trial of administration of MSC to patients with respiratory distress type COVID-19	Spain, Madrd	Adipose Tissue	50 Control 50 Exp
IRCT20140911019125N6	The effect of dental pulp mesenchymal stem cells in treatment of corona disease	Iran	Dental Pulp	10 Exp
IRCT20140528017891N8	The effect of stem cell transplantation in the treatment of COVID-19	Iran	Umbilical Cord	10 Exp
IRCT20200325046860N2	Mesenchymal Stem Cell therapy in COVID19	Iran	Not Specified	5 Exp
IRCT20200217046526N1	Mesenchymal Stem Cell Therapy for Acute Respiratory Distress Syndrome in Coronavirus Infection	Iran	Not Specified	6 Exp
NCT03042143	Repair of Acute Respiratory Distress Syndrome by Stromal Cell Administration (REALIST) (COVID-19) (REALIST), Phase I/II	UK, Belfast	Umbilical Cord	9 Phase I 33 Control 33 Exp
NCT04293692	Therapy for Pneumonia Patients iInfected by 2019 Novel Coronavirus	China, Hubei	Umbilical Cord	12 Control 12 Exp
NCT04352803	Adipose Mesenchymal Cells for Abatement of SARS-CoV-2 Respiratory Compromise in COVID-19 Disease	USA + Spain	Adipose Tissue	10 Control 10 Exp
NCT04366830	Intermediate-size Expanded Access Program (EAP), Mesenchymal Stromal Cells (MSC) for Acute Respiratory Distress Syndrome (ARDS) Due to COVID-19 Infection	USA, New York	Not Specified	50 Exp
EUCTR2020-001364-29-ES	Study with stem cells from allogenic adipose tissue, in patients with coronavirus severe pneumonia	Spain, Seville	Adipose Tissue	13 Control 13 Exp
EUCTR2020-001505-22-ES	Efficacy and safety evaluation of umbilical cord mesenchymal stem cells for the treatment of patients with respiratory failure due to coronavirus (COVID-19)	Spain, Barcelona	Umbilical Cord	15 Control 15 Exp
NCT04348435	A Randomized, Double-Blind, Placebo-Controlled Clinical Trial to Determine the Safety and Efficacy of Hope Biosciences Allogeneic Mesenchymal Stem Cell Therapy (HB-adMSCs) to Provide Protection Against COVID-19	USA, Texas	Adipose Tissue	50 Control 50 Exp
NCT04355728	Use of UC-MSCs for COVID-19 Patients	USA, Florida	Umbilical Cord	12 Control 12 Exp
NCT04361942	Treatment of Severe COVID-19 Pneumonia With Allogeneic Mesenchymal Stromal Cells (COVID_MSV) COVID_MSV	Spain, Valladolid	Not Specified	12 Control 12 Exp
NCT04366063	Mesenchymal Stem Cell Therapy for SARS-CoV-2-related Acute Respiratory Distress Syndrome	Iran	Not Specified	20 Control 20 Exp 1 20 Exp 2
NCT04371601	Safety and Effectiveness of Mesenchymal Stem Cells in the Treatment of Pneumonia of Coronavirus Disease 2019	China, Fujian	Umbilical Cord	30 Control 30 Exp
NCT04377334	Mesenchymal Stem Cells (MSCs) in Inflammation-Resolution Programs of Coronavirus Disease 2019 (COVID-19) Induced Acute Respiratory Distress Syndrome (ARDS)	Germany	Bone Marrow	20 Control 20 Exp
IRCT20200413047063N1		Iran		10 Control 10 Exp
IRCT20200418047121N2	Stem cell therapy in COVID-19	Iran		3 Control 3 Exp
NCT04366271	Clinical Trial of Allogeneic Mesenchymal Cells From Umbilical Cord Tissue in Patients With COVID-19 MESCEL-COVID19	Spain, Madrid	Umbilical Cord	53 Control 53 Exp
NCT04390139	Efficacy and Safety Evaluation of Mesenchymal Stem Cells for the Treatment of Patients With Respiratory Distress Due to COVID-19 COVIDMES	Spain, Barcelona	Umbilical Cord	15 Control 15 Exp
NCT04390152	Safety and Efficacy of Intravenous Wharton's Jelly Derived Mesenchymal Stem Cells in Acute Respiratory Distress Syndrome Due to COVID 19	Colombia	Umbilical Cord	20 Control 20 Exp
NCT04392778	Clinical Use of Stem Cells for the Treatment of Covid-19	Turkey	Not Specified	15 Control 15 Exp
NCT04400032	Cellular Immuno-Therapy for COVID-19 Acute Respiratory Distress Syndrome—Vanguard CIRCA-19	Canada	Bone Marrow	3 Exp1 3 Exp 2 3 Exp 3
ACTRN12620000612910	The MEND (MEseNchymal coviD-19) Trial: a pilot study to investigate early efficacy of mesenchymal stem cells in adults with COVID-19	Australia	Mesenchymo-Angioblast	12 Control 12 Exp
NCT04382547	Treatment of Covid-19 Associated Pneumonia With Allogenic Pooled Olfactory Mucosa-derived Mesenchymal Stem Cells	Belarus	Olfactory-Mucosa	20 Control 20 Exp
NCT04397796	Study of the Safety of Therapeutic Tx With Immunomodulatory MSC in Adults With COVID-19 Infection Requiring Mechanical Ventilation	USA, California	Bone Marrow	23 Control 23 Exp
NCT04416139	Mesenchymal Stem Cell for Acute Respiratory Distress Syndrome Due for COVID-19 COVID-19	Mexico	Umbilical Cord	5 Control 5 Exp
IRCT20200421047150N1	Stem cell treatment for COVID-19	Iran	Umbilical Cord	45 Control 45 Exp
IRCT20160809029275N1	stem cell therapy in Covid-19	Iran	Umbilical Cord	10 Control 10 Exp
NCT04366323	Clinical Trial to Assess the Safety and Efficacy of Intravenous Administration of Allogeneic Adult Mesenchymal Stem Cells of Expanded Adipose Tissue in Patients With Severe Pneumonia Due to COVID-19	Spain, Andalucia	Adipose Tissue	13 Control 13 Exp
NCT04371393	MSCs in COVID-19 ARDS	USA, New York	Not Specified	150 Control 150 Exp
NCT04399889	hCT-MSCs for COVID19 ARDS	USA, North Carolina	Umbilical Cord	15 Control 15 Exp
NCT04428801	Autologous Adipose-derived Stem Cells (AdMSCs) for COVID-19	USA, Texas	Adipose Tissue	100 Control 100 Exp
NCT04429763	Safety and Efficacy of Mesenchymal Stem Cells in the Management of Severe COVID-19 Pneumonia CELMA	Colombia	Umbilical Cord	15 Control 15 Exp
ISRCTN33578935	To study the treatment of COVID-19 with severe viral pneumonia by using purified stem cell exosomes	Germany		32 Control 32 Exp
NCT04389450	Double-Blind, Multicenter, Study to Evaluate the Efficacy of PLX PAD for the Treatment of COVID-19	USA, California		70 Control 70 Exp

*Exp* experimental

## Conclusions

As the world waits for the development and ready availability of an effective vaccine against SARS-CoV-2, there is also an anticipation to learn of the outcomes of the ongoing trials utilising MSCs as a cellular therapy to combat the effects of this virus. With no specific drug against SARS-CoV-2 or its effects available, there is great hope that MSCs will provide a means to curtail the aggressive inflammatory response seen in patients severely affected by COVID-19. While the early published pilot studies have provided a promising outlook for this strategy, data from better powered and controlled trials will be needed to make more conclusive judgments.

## Data Availability

Not applicable.

## Abbreviations

ACE2: Angiotensin converting enzyme 2
ARDS: Acute Respiratory Distress Syndrome
BALF: BronchoAlveolar Lavage Fluid
BM: Bone Marrow
CCR: Chemokine Receptor
CD: Cluster of Differentiation
COVID-19: Coronavirus Disease 19
CT: Computer Tomography
CXCL: Chemokine (C-X-C motif) Ligand
CXCR: Chemokine (C-X-C motif) Receptor
HLA: Human Leukocyte Antigen
IDO1: Indoleamine-Pyrrole 2,3-Dioxygenase 1
IL: Interleukin
INF-γ: Interferon gamma
MERS: Middle East Respiratory Syndrome
MSC: Mesenchymal Stromal Cell
NK Cells: Natural Killer Cells
PGE2: Prostaglandin E2
RBD: Receptor Binding Domain
RNA: Ribonucleic Acid
SARS: Severe Acute Respiratory Syndrome
SARS-CoV: Severe Acute Respiratory Syndrome– Coronavirus
SARS-CoV-2: Severe Acute Respiratory Syndrome – Coronavirus 2
TGF-β: Transforming Growth Factor beta
TLR: Toll Like Receptor
TMPRSS2: Transmembrane protease, serine 2
TNFα: Tumour Necrosis Factor alpha
UC: Umbilical Cord
WHO: World Health Organisation
